# Silence=Death Redux: Infectious Diseases, Public Health, and the Imperative to Resist

**DOI:** 10.1093/cid/ciaf155

**Published:** 2025-03-24

**Authors:** Eric A Meyerowitz, Ilan S Schwartz

**Affiliations:** Department of Medicine, Division of Infectious Diseases, Montefiore Medical Center, Bronx, New York, USA; Department of Medicine, Division of Infectious Diseases, Albert Einstein College of Medicine, Bronx, New York, USA; Department of Medicine, Division of Infectious Diseases, Duke University, Durham, North Carolina, USA

**Keywords:** activism, advocacy, policy, HIV, Trump

## Abstract

The field of infectious diseases is at a perilous moment in our history, with the United States executive branch of government and congressional enablers contemptuous toward truth, public health, and social justice, implementing regressive policies that promise to undo decades of scientific and social progress and directly threaten the lives of individuals from vulnerable communities that we serve around the world. In this perspective, we recount the early executive actions that impact every aspect of the field infectious diseases. Inspired by LGBTQ+ community activists and early human immunodeficiency virus (HIV) clinicians who overcame hostile political forces, we implore the infectious diseases community to urgently and forcefully defend the people for whom we care, the public health institutions on which we rely, and the principles in which we believe.

## Teaser

The second Trump administration has released a torrent of executive policies hostile to public health, science, and marginalized populations. We outline the vast harms of these policies and call for urgent action and resistance.

In the relatively brief history of the medical specialty of infectious diseases (ID), at least 3 seismic events have transformed not just our field, but the world as we know it: the emergence of human immunodeficiency virus (HIV), the coronavirus disease 2019 (COVID-19) pandemic, and the second Trump presidency. In the face of the immense devastation caused by the former 2 plagues, ID clinicians quickly learned that effectively addressing them required us to step outside our clinical roles and provide clear, compelling, and uncompromising advocacy for just and scientifically informed policies [1, 2]. Similarly, the Trump administration's full-scale attack on science, public health, and social justice demands that we urgently find our voices and gather our strength to forcefully defend these principles.

The return of Donald Trump to the United States Presidency has brought a seemingly endless torrent of injustice. Soon after taking office, Trump signed a barrage of executive orders that have plunged the country into chaos and immediately and severely damaged our ability to prevent outbreaks and provide care for persons with infections. With the stroke of a pen, America was withdrawn from the World Health Organization [3], hamstringing pandemic preparedness; protections for undocumented immigrants (including the sanctity of hospitals, along with schools and houses of worship) were abolished [4], leading to interruptions in care for some of our most vulnerable patients; hostility to transgender and gender-diverse persons was codified [5]; scientists at public health agencies were muzzled, prevented from communicating with state and international counterparts, clinicians, or the public; Center for Disease Control and Prevention [CDC] publications, including *Emerging Infectious Diseases* and *Morbidity and Mortality Weekly Report*, were halted [6], impeding clinician awareness of emerging infectious risks; federally housed data and guidance documents were taken offline and sanitized, replaced with revisions that erased the existence of vulnerable populations (like transgender women, who were scrubbed from recommendations for mpox vaccination [7, 8]), and thousands of scientists and epidemiologists working at the National Institutes of Health (NIH) and CDC were summarily dismissed [9]. The United States Agency for International Development (USAID) was dismantled [10], and humanitarian aid was frozen, leading to immediate cessation of hundreds of active programs that prevent and control outbreaks, develop new biomedical interventions, and provide care for millions with infections. Antiretroviral distribution through Presidential Emergency Plan For AIDS Relief (PEPFAR) was suspended, leading to treatment interruptions for millions of people with HIV, with modeling projecting more than half a million new HIV infections in South Africa alone over the next decade and years shaved from life-spans for people with HIV [11]. Additionally, funding for the NIH was deeply cut, threatening the financial viability of academic medical centers and research universities, which will damage the clinician-scientist pipeline [12, 13], and deprive future patients of benefits from biomedical advances. And that was only the first 3 weeks in office.

The field of ID came of age during the HIV epidemic. From brave patients, community organizers, and trailblazing clinicians, we have learned the hard way of the need for advocacy. For decades, members of our profession have cared for people marginalized by society—immigrants, gay and bisexual men, transgender and gender-diverse individuals, women, and people of every race or spoken language. Each time we look into the eyes or place our stethoscope on the chest of someone suffering indignities of discrimination, we are affirming their value and importance to our world. We have seen how societal and structural forces like racism, misogyny, homophobia, transphobia, and xenophobia contribute to worse health outcomes [14]. We have learned about the importance of countering such forces through self-affirming and judgement-free care and thoughtful, evidence-based public policy, such as Ryan White Program-funded comprehensive care clinics, where people with HIV can receive substance abuse counseling and social work support alongside medical care [15–17]. We also learned—primarily from the organization and mobilization of patient groups and community activists, like AIDS Coalition to Unleash Power (ACT UP) [18] or South Africa's Treatment Action Campaign [19]—the importance of joining together to rise up against injustice and helping advance good policy for our patients. These lessons were echoed during the COVID-19 pandemic, when clinicians collaborated with epidemiologists, engineers, patient groups, and concerned citizens, among others, to challenge misinformation and advocate for policies that were sound and just [2].

Throughout the Trump administration's onslaught, the silence from medical societies and institutions has been deafening. Some societies have rejected calls from their members to speak out against the administration's policies, framing this as an affront to their bipartisan mission and preferring instead to work with the administration and aligned legislators through diplomacy rather than risk exposure to retribution. However, we believe that they have misjudged the situation. The actions of the Trump administration and congressional enablers make clear that their goal is not to improve an imperfect society but to destroy and fundamentally rebuild one based on contempt for egalitarianism, truth, and justice. For example, the confirmation of Robert F. Kennedy, Jr, an antivaccine activist [20] who has repeatedly lied about the safety and efficacy of vaccines and medicines, lent credence to HIV denialism, and embraced racist pseudoscience, to lead the Department of Health and Human Services will increase distrust of and possibly impede access to life-saving vaccines and other biomedical interventions. That an individual so deeply unfit to run the nation's healthcare apparatus was nominated and confirmed should vanquish any illusions of the existence of good faith and reason that would be required for finding a common ground.

To meet this moment, to survive as a field, and to serve our patients and society, the only way forward is to stand together and speak out continuously and forcefully. We must speak for those who cannot: muzzled colleagues employed by federal agencies, vulnerable patient populations, and future generations. The challenges are many, but we—and our professional societies—can start with a few concrete actions:

First, we need to reaffirm to our colleagues, trainees, and patients our steadfast and immovable commitment to the bedrock principles of inclusion, diversity, access, and equity (IDA&E), core values adopted by the Infectious Diseases Society of America in 2018 [21]. We must unequivocally reject any implication that healthcare professionals from underrepresented backgrounds are any less qualified; on the contrary, a diverse ID workforce is essential to our collective goal of accessible, culturally competent, and trusted care for the disparate communities we serve [21]. We need to redouble efforts to recruit and retain individuals underrepresented in medicine and improve access to ID expertise for all [22, 23]. We must make clear to our patients and communities that we will continue to care for them irrespective of their background, gender identity, immigration status, or political convictions, and that we will continue to advocate for accessible care and just policies.

Second, we need to ensure that trainees and colleagues are protected from targeted harassment for advocacy or providing the best care for our patients. We must be cognizant that our aspiration to provide nationwide access to ID clinicians may mean heightened vulnerability for those practicing in rural areas, and we must commit to supporting these individuals.

Third, we must forcefully defend science and evidence-based care, and support scientifically informed policy that benefits our patients and communities. We must clearly convey to policy makers how actions that impede science or undermine public health will directly imperil the health and economic wellbeing of the nation and/or their constituents. Moreover, we must impress on the public how harmful policies are expected to affect them and their immediate communities. We must oppose pseudoscience and intensify our efforts to engender trust in safe and proven interventions, like vaccines, pre-exposure prophylaxis to prevent HIV infection, and gender-affirming care.

Fourth, we must call out and vigorously oppose censorship, including prescribed revision of science-based communications based on ideological grounds. For example, we cannot abide by government efforts to erase websites, scrub inconvenient words, muzzle scientists and public health personnel, deny publication or online dissemination of peer- (not politician-) reviewed scientific outputs, or interject inaccurate and discriminatory language into our scientific and clinical work. We need to take measures to ensure timely data and clinical guidance are available, whenever and wherever needed. Where federal agencies are prevented from hosting data sets or clinical practice documents, medical societies or universities or individual states should fill in.

Fifth, we need to settle in for a long fight. While mounting an urgent response, we also must recognize the chronicity of the threat and plan accordingly. We should ensure that ID trainees and early career faculty are prepared through modeled behavior and training [24] to resist structural inequality and harmful policies. We must also remain focused on longstanding goals like ending the HIV epidemic, eliminating hepatitis C virus infections and their sequelae, eradicating maternal-fetal infections, combating antimicrobial resistance, improving outbreak preparedness, and reducing suffering from infections.

In conclusion, public health, science, and our patients are under attack, and our allegiances are being tested. In Camus's *The Plague*, Jean Tarrou notes: “All I maintain is that on this earth there are pestilences and there are victims, and it's up to us, so far as possible, not to join forces with the pestilences” [25]. Now we, too, must fight the scourges of pathogens, intolerance, and harmful, regressive policy to defend science, global and domestic public health, and our patients. There is no middle ground: obedience is complicity. As we were humbly taught by brave LGBTQ+ community activists who decades ago fought stigma and injustice amid a raging HIV epidemic and a hostile political environment, Silence = Death.

## Notes

***Financial support**.* No funding was received for this manuscript.

***Potential conflicts of interest**.* The authors have no conflicts to disclose. All authors have submitted the ICMJE Form for Disclosure of Potential Conflicts of Interest.
